# Effect of Different Durations of Adjuvant Capecitabine Monotherapy on the Outcome of High-Risk Stage II and Stage III Colorectal Cancer: A Retrospective Study Based on a CRC Database

**DOI:** 10.3390/curroncol30010072

**Published:** 2023-01-10

**Authors:** Qiao Yu, Zhigui Li, Yuqing Liu, Yichen Luo, Jingya Fan, Peijun Xie, Xiaoman Cao, Xingyu Chen, Xiaodong Wang

**Affiliations:** 1 Department of Gastrointestinal Surgery, Department of General Surgery, West China Hospital of Sichuan University, Chengdu 610041, China; 2 West China School of Medicine, West China Hospital of Sichuan University, Chengdu 610041, China

**Keywords:** capecitabine, monotherapy, colorectal cancer, adjuvant chemotherapy

## Abstract

(1) Background: The duration of adjuvant chemotherapy recommended by the NCCN guidelines is 6 months. However, patients are not compliant with intravenous chemotherapy for many reasons; therefore, one approach is to obtain a survival benefit by prolonging the duration of capecitabine monotherapy. (2) Methods: A total of 355 qualified colorectal cancer (CRC) patients from January 2010 to December 2020 at West China Hospital of Sichuan University were selected to receive capecitabine monotherapy for 6–9 months and >12 months. The main endpoints were overall survival (OS) and disease-free survival (DFS). (3) Results: Among stage III patients, in the >12 months (12M) and 6–9 months (6M) groups, the 5-year DFS rates were 80.7%% and 66.8%, respectively, and the 5-year OS rates were 94.7%% and 88.8%, respectively. Among high-risk stage II patients, in the >12 months (12M) and 6–9 months (6M) groups, the 5-year DFS rates were 81.5% and 78.6%, respectively, and the 5-year OS rates were 93.1% and 84.2%, respectively. (4) Conclusions: Twelve months of chemotherapy demonstrated superior OS and DFS to that of six months in the stage III group but showed no difference in the high-risk stage II group. The better OS and DFS observed in the 12-month treatment period could be of value in selected cases.

## 1. Introduction

Colorectal cancer (CRC) accounts for 10% of all cancer diagnoses and cancer-related deaths worldwide. It is the second most common cancer among women and the third most common cancer among men [1]. Patients with pathological TNM stage II-III CRC account for the majority of CRC patients, and patients with pathological stage II-III CRC have a worse prognosis than patients with pathological stage 0-I CRC [2].

For patients with pathological TNM stage II-III CRC, the recommended treatment is adjuvant chemotherapy after tumor resection. To date, adjuvant chemotherapy regimens based on fluorouracil (fluoropyrimidines Fp) and/or oxaliplatin [3,4] are widely considered to improve the long-term prognosis of patients with CRC [3,4,5,6]. The drugs used in different adjuvant chemotherapy regimens all vary in the way they are administered, with chemotherapy regimens such as CAPOX and FOLFOX in combination with oxaliplatin requiring patients to visit a medical service center for chemotherapy with intravenous drip, while capecitabine monotherapy allows patients to finish adjuvant chemotherapy at home.

Capecitabine has fewer toxic reactions than 5-Fu/LV [7], is easier to administer, and is associated with cost-effectiveness compared to intravenous chemotherapy without oxaliplatin [8], which suggests the possibility of extending the duration of chemotherapy. As mentioned above, many studies have demonstrated that a 6-month combination adjuvant chemotherapy regimen has a better prognosis than 6-month capecitabine monotherapy for patients with stage II-III CRC [5]. However, we found that some patients have difficulty or are unwilling to go to medical centers to complete adjuvant chemotherapy regimens because of natural disasters, including the ongoing COVID-19 pandemic [9], or other kinds of public health challenges. In China and many other countries, patients’ financial conditions, distance from medical centers, and their willingness should also be considered. In addition, a substantial proportion of patients are not compliant with intravenous chemotherapy [10]. For these groups of patients, the only solution is to receive capecitabine monotherapy if they want chemotherapy. Additionally, we found that there is another group of patients who tend to actively request that their physicians prolong adjuvant chemotherapy, which may cause stronger toxic effects with no improvement in survival [11]. There are already relevant phase III clinical studies investigating the prognosis of prolonged oral monotherapy [12]. In the present study, we investigate whether extending the duration of oral monotherapy with capecitabine in Chinese patients with high-risk stage II and stage III CRC will lead to better outcomes and provide a reference for the dosing strategy of oral monotherapy with capecitabine.

## 2. Materials and Methods

### 2.1. Study Design

This was a unicentral, retrospective study. The study was conducted in accordance with the Declaration of Helsinki and the Ethical Guidelines for Clinical Research in China, and it was approved by the Ethics Committee on Biomedical Research, West China Hospital of Sichuan University (No. 2021-1773). The study was a retrospective trial and did not involve patient interventions; thus, informed consent was not required for the study.

In this study, all the patients who received capecitabine monotherapy were divided into high-risk stage II [4] patients and stage III patients according to TNM staging. In each group, we divided the participants into two groups: the 12M group, which received capecitabine monotherapy for more than 12 months, and the 6M group, which received capecitabine monotherapy from 6 to 9 months. It has been proven in many clinical practices that prolonging the duration of adjuvant treatment will not decrease the survival of patients as long as it does not bring additional toxic reactions.

### 2.2. Eligibility Criteria

All patients met the following criteria: (1) 18 years of age or older; (2) complete baseline information; (3) well-preserved main organ functions; (4) a pathologically confirmed diagnosis of colon or rectal cancer (adenocarcinoma); (5) underwent total mesenteric resection (TME) or complete mesorectal excision (CME); (6) clinical stage III or high-risk stage II (high-risk factors for recurrence (exclusive of those cancers that are MSI-H): poorly differentiated/undifferentiated histology, lymphatic/vascular invasion, bowel obstruction, <12 lymph nodes examined, perineural invasion, localized perforation, or close, indeterminate, positive margins, or tumor budding) [4]; (7) R0 resection on postoperative pathology; (8) postoperative capecitabine monotherapy regimen; (9) no change in chemotherapy regimen during chemotherapy; (10) mismatch repair was not included in the analysis of this trial.

### 2.3. Protocol Treatment

Capecitabine was administered orally as a single agent at standard doses, with all patients receiving 1250 mg/m^2^ in the morning and in the evening with meals for 14 days, followed by a 7-day break. It was a 21-day course of treatment.

In this retrospective cohort, patients could discontinue the treatment under the following situations: (1) severe toxic reaction or grade >3 adverse events (AEs); (2) disturbance to their quality of life; (3) recurrence of the primary disease; (4) certain situations in which the attending physician felt the treatment should be ended, for example, the occurrence of comorbidities; (5) transfer to another hospital.

### 2.4. Primary/Secondary Endpoint and Statistical Analysis

#### 2.4.1. Primary/Secondary Endpoint

The primary endpoint was overall survival (OS), which was defined as survival from registration to death out of any cause. The patients were censored if no events occurred at the final data cutoff. The secondary endpoint was disease-free survival (DFS), which was defined as the survival from registration to (1) death from any cause, (2) recurrence, and (3) occurrence of any cancer.

#### 2.4.2. Statistical Analysis

SPSS Statistics 26.0 (IBM, Armonk, NY, USA) and R studio were used for statistical analysis. DFS and OS were used for survival analysis, and survival time was measured in days. The *t*-test was used for the comparison of sample means between different adjuvant chemotherapy regimens in the same cohort, ANOVA was used for the comparison of sample means in multiple groups in parametric statistics, the X^2^ test was used for the comparison of rates, and the Spearman test was used for correlation analysis. The Kaplan–Meier method was used for graphs to compare DFS and OS in different groups in the same cohort, and Cox proportional hazards model analysis was performed to explore the factors affecting the 5-year disease-free survival and overall survival of patients. The hazard ratio (HR) and two-tailed 95% confidence interval (CI) were used to estimate the relative risk of overall survival and disease-free survival between different adjuvant chemotherapy regimens in the same cohort. A two-tailed *p* < 0.05 was considered statistically significant, and *p*-values were corrected according to the Bonferroni method when multiple groups were involved.

## 3. Results

### 3.1. Patient Characteristics

All patients were enrolled from a CRC database [13] of the Gastrointestinal Surgery Center of West China Hospital of Sichuan University. A total of 355 patients with CRC from January 2010 to December 2020 at West China Hospital of Sichuan University were selected and distributed into two groups via their TNM staging (AJCC 8th), including 216 patients (61%) with high-risk stage II CRC and 139 patients (39%) with stage III CRC. All patients in this study had good baseline balance (*p* > 0.05). The baseline information of the patients is shown in Table 1. According to TNM staging, 355 patients who received capecitabine monotherapy were divided into high-risk stage II patients and stage III patients, 221 and 147 patients, respectively. The median duration of chemotherapy was 7.6 months in the 6M group, ranging from 6.0 to 9.0 months, and the median duration of chemotherapy was 15.6 months in the 12M group, ranging from 12 to 49.3 months.

### 3.2. Disease-Free Survival

DFS was based on 5.25 years of median follow-up.

**Figure 1:**
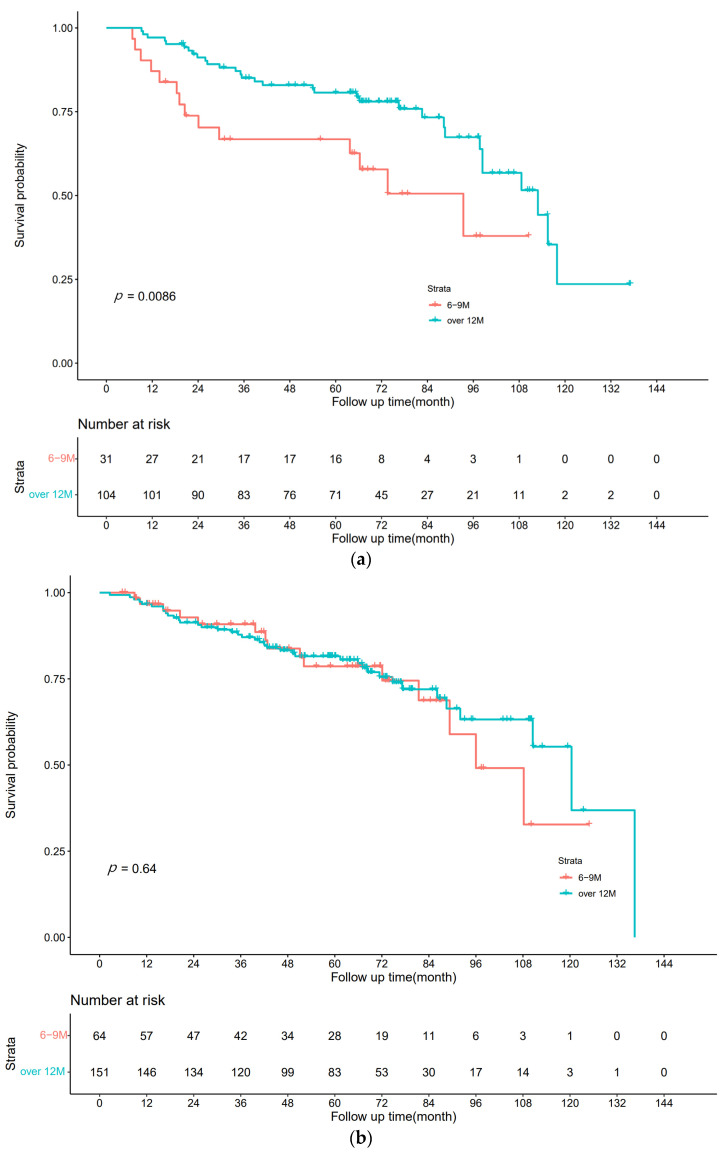
DFS. (**a**) Stage III CRC; (**b**) high-risk stage II CRC.

Among 135 stage III patients, the 12M arm containing 104 patients had a higher 5-year DFS rate of 80.7% (95% CI: 76.7%–84.7%) than the 6M arm containing 31 patients at 66.8% (95% CI: 58.2%–75.4%). K-M curves were plotted in Figure 1a. Multivariate analysis using Cox proportional hazards regression revealed that >12 months of monotherapy improved DFS, with HR = 0.431 (95% CI: 0.226–0.821) *p* = 0.011.

Among 215 high-risk stage II patients, the 5-year DFS rate of 81.5% (95% CI: 78.2%–84.8%) in the 12M group containing 151 patients was the same as that in the 6M group containing 64 patients at 78.6% (95% CI: 72.5%–84.7%). K-M curves were plotted as shown in Figure 1b. Multivariate analysis using Cox proportional hazards regression yielded HR = 0.917 (95% CI: 0.501–1.679), *p* = 0.778, with no significant difference between the two groups.

The results showed that extending capecitabine monotherapy beyond 12 months (16 cycles) compared to the conventional 6 months (8 cycles) of capecitabine monotherapy resulted in a benefit in disease-free survival in patients with stage III CRC but did not increase DFS in patients with high-risk stage II CRC.

### 3.3. Overall Survival

OS was based on 5.52 years of median follow-up.

**Figure 2:**
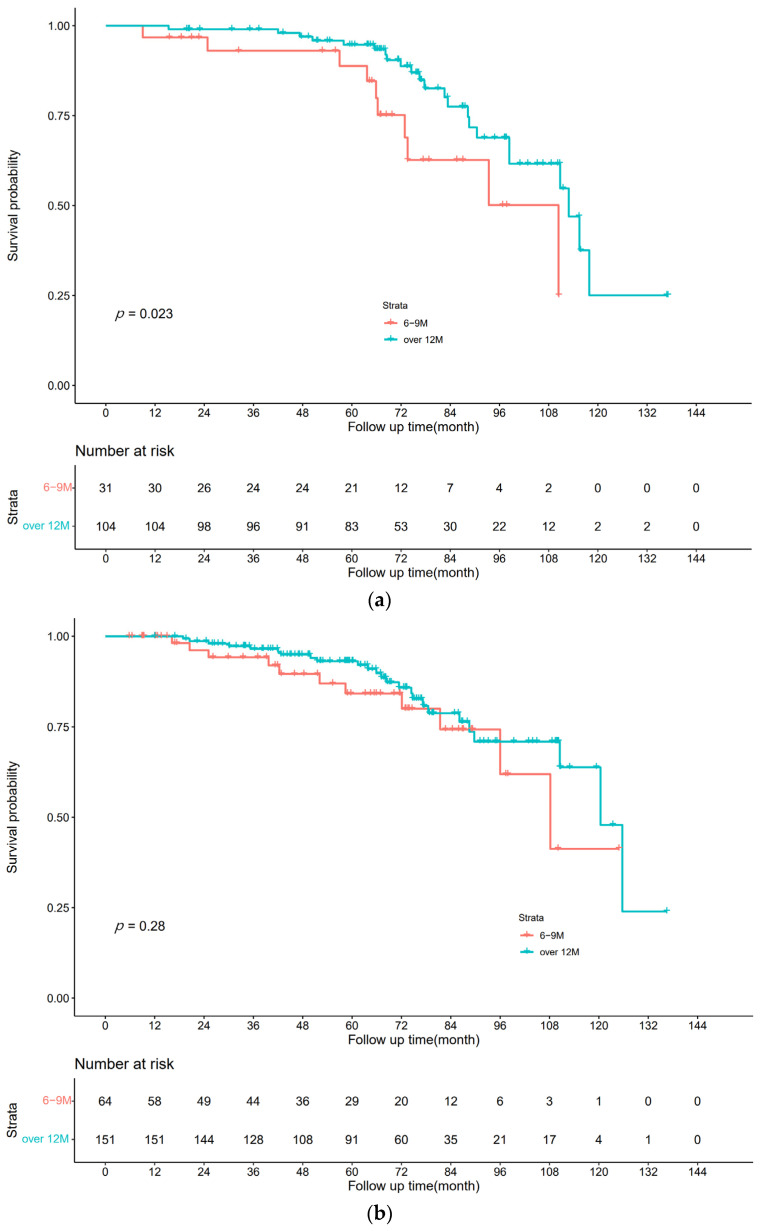
OS. (**a**) Stage III CRC; (**b**) high-risk stage II CRC.

Among all 135 stage III patients, the 12M arm containing 104 patients had a higher 5-year OS rate of 94.7% (95% CI: 92.4%–97.0%) than the 6M arm containing 31 patients at 88.8% (95% CI: 82.7%–94.9%). The K-M curves are plotted in Figure 2a. Multivariate regression analysis using Cox proportional hazards regression revealed that >12 months of monotherapy improved OS, with HR = 0.420 (95% CI: 0.194–0.906), *p* = 0.027.

Among 215 high-risk stage II patients, the 5-year OS rate of 93.1% (95% CI: 90.9%–95.3%) in the 12M group containing 151 patients was the same as that in the 6M group containing 64 patients at 84.2% (95% CI: 78.6%–89.8%). The K-M curves are plotted in Figure 2b. Multivariate regression analysis using Cox proportional hazards regression yielded HR = 0.733 (95% CI: 0.354–1.517), *p* = 0.403, with no significant difference between the two groups.

The results showed that extending capecitabine monotherapy beyond 12 months (16 cycles) compared to the conventional 6 months (8 cycles) of capecitabine monotherapy resulted in an overall survival benefit in patients with stage III CRC; however, it had no benefit on OS in patients with high-risk stage II CRC.

## 4. Discussion

### 4.1. Prognostic Impact of Different Durations of Capecitabine Monotherapy

In the X-ACT [14] trial carried out in 2005, researchers found that capecitabine is equal to the earlier 5-Fu/LV-based chemotherapy, and it showed a decrease in adverse events compared to 5-Fu/LV. Several similar studies were carried out [7], and they found that capecitabine is equal to 5-Fu/LV in CRC. Since then, capecitabine has been recommended in many clinical guidelines as monotherapy for patients who are not compliant with intravenous chemotherapy or are facing severe side effects when undergoing an intravenous chemotherapy regimen. Additionally, a systematic review from Fiona [7] revealed that participants treated with oral fluoropyrimidines experienced less grade ≥ 3 neutropenia/granulocytopenia, stomatitis, and any other grade ≥ 3 AEs, but it brought about more grade ≥ 3 hand–foot syndrome (HFS). In a previous RCT [12] (JFMC37-0801) comparing 6 months of monotherapy with 12 months of monotherapy, the incidence rates of AEs in the 6-month group and 12-month group were 91.7% and 94.7%, respectively, and the most common AE was hand–foot syndrome (HFS). The 12-month group experienced a higher cumulative incidence of HFS than the standard 6-month group, and even in the 12-month group, the toxicities were still clinically acceptable. A preplanned safety analysis indicated that prolonging capecitabine monotherapy from 6 months to 12 months does not result in an unacceptable increase in severe toxic reactions [15].

Many studies on the duration of adjuvant chemotherapy for CRC have been conducted in the past [16], and a 6-month postoperative 5-Fu-based adjuvant chemotherapy regimen was established based on such studies, followed by the use of oral 5-Fu-based chemotherapy in combination with oxaliplatin, resulting in first-line chemotherapy regimens such as CAPOX and FOLFOX, which are now recommended in guidelines worldwide. However, chemotherapy regimens with the addition of oxaliplatin have resulted in an increase in neurotoxicity caused by oxaliplatin compared to monotherapy based on 5-Fu, especially in the latter portion of the treatment period. To reduce the toxic effects associated with the increase in chemotherapy duration, studies now focus on the near-term and long-term efficacy of 3-month versus 6-month CAPOX/FOLFOX chemotherapy, with IDEA studies [17] pooling data from multiple centers. Although the final study did not yield noninferiority results at the aggregate level as expected, a subgroup analysis found that 3 months of FOLFOX/CAPOX was adequate in the low-risk subgroups of T1-3 and N1, while in the high-risk subgroups of T4 or N2, patients treated for the standard duration of chemotherapy had significantly improved OS and DFS compared with the 3-month chemotherapy.

In a large phase III clinical trial [12] (JFMC37-0801) examining the efficacy of 6 months of capecitabine monotherapy versus 12 months of capecitabine monotherapy in patients with stage III colon cancer, Naohiro demonstrated that patients treated with 12 months of oral monotherapy had significantly higher OS and RFS than patients treated with 6 months of oral monotherapy, but the difference in DFS between the two groups was not significant. Thus, they conducted a subgroup analysis to determine the differentiation in DFS between these two kinds of treatment, which also defined a lower-risk group of patients with T1-3 and N1 disease and a high-risk group with T4, N2, or both. They found a significant difference in the hazard ratio in the high-risk group, which agreed with the outcome of the IDEA trial in 2017. In 2015, Sadahiro [18] also conducted a trial that evaluated the difference between 6 months and 18 months of oral monotherapy but failed to show the superiority of 18 months of adjuvant chemotherapy over a 6-month regimen. To the best of our knowledge, there are no other studies with the same purpose or details as ours.

However, the above discussion on monotherapy does not mean that it can replace combined chemotherapy, regardless of whether it is prolonged to 12 months. Until now, according to the NCCN guidelines, the first-line adjuvant chemotherapy regimens for stage III and high-risk stage II colorectal cancer are 6 months of XELOX/FOLFOX and 6 months of capecitabine monotherapy, respectively. Previous studies have shown that combined chemotherapy, such as XELOX/FOLFOX, has a better therapeutic outcome than oral monotherapy [4]. Therefore, we conducted this study to find an alternative therapy for those patients who do not want combined chemotherapy for any reason.

In this study, we compared the advantages and disadvantages of oral monotherapy based on capecitabine for 6 to 9 or >12 months in patients with high-risk stage II and stage III CRC, respectively, and found that in high-risk stage II patients, the prolongation of oral monotherapy did not result in an increase in OS and DFS. The hazard ratio showed no significance regarding DFS and OS: HR = 0.917 (95% CI: 0.501–1.679), *p* = 0.778; HR = 0.733 (95% CI: 0.354–1.517), *p* = 0.403, indicating that 6 months of capecitabine monotherapy is sufficient for high-risk stage II patients. The result was compliant with the NCCN guidelines where, although oral monotherapy is recommended for high-risk stage II patients, its duration should not exceed 6 months [7]. Our results revealed that for patients with stage III CRC, the difference in DFS and OS was significant between >12 months of capecitabine monotherapy and 6 months of capecitabine monotherapy. In all, our result was consistent with the IDEA study, where patients with a lower risk of death do not benefit from higher chemotherapy intensity or longer chemotherapy duration.

### 4.2. Limitations of the Study

This study has several limitations. First, it was a retrospective cohort study, and the patients selected were well-balanced at baseline (*p* > 0.05), but the selection of patients remained prone to bias. Second, the follow-up interval of the database used in this paper was primarily 1–3 months, and the longer the follow-up period of the patients, the longer the follow-up interval. Thus, the patients themselves or their family members were often required to recall the time of the patients’ relevant symptoms; thus, there was a corresponding recall bias. Third, because of the lack of relevant information and clinical record deficiencies, the study did not include the molecular biology or genetic characteristics related to the patients’ diseases, and the patients’ toxic reactions and compliance were not assessed. Fourth, the follow-up period of the patients was short, with a median follow-up period of only 5.52 years, and a longer follow-up period might have yielded more accurate results. Fifth, this study was not compared with the chemotherapy regimen recommended by the current guidelines at the time of experimental design, but the study itself was based on value-based medicine, which meant that it was guided by the greatest benefit to patients. This study was aimed at patients who could not or did not want to receive standard adjuvant chemotherapy by intravenous injection. Sixth, the patients included in the study spanned 10 years, and the bias brought about by different laboratory or imaging standards and different socioeconomic environments within the 10 years could not be ignored. Seventh, because of the practice of combined chemotherapy, the population of patients who received capecitabine monotherapy was not sufficient, although we achieved a significant outcome.

### 4.3. Practical Significance of This Research

It seems that the extended duration of chemotherapy is contrary to the existing guideline recommendations. However, we should consider not only the patient’s own desire for a higher chance of survival but also the patient’s difficulty in following the guideline-recommended chemotherapy regimen with intravenous involvement for their own or external reasons. In China, there is a significant population that lives so far from a medical center that they need to spend two days traveling to receive an IV chemotherapy treatment. A long journey is likely to be torturous for the patient, and this is a common complaint among patients. At the same time, there is another population who knows the survival benefit of intravenous chemotherapy but has poor compliance with intravenous adjuvant chemotherapy and still prefers to take oral monotherapy. This study was only conducted with patients with high-risk stage II and stage III CRC who refused intravenous chemotherapy for their own or external reasons. Our aim was to confirm for these patients whether their treatment should be prolonged based on monotherapy. Additionally, previous trials have shown that prolonging capecitabine monotherapy from 6 months to 12 months does not result in an unacceptable increase in severe toxic reactions [15]. Regarding cost-effectiveness, Heditishin [19] conducted an antecedent study of JFMC37-0801, which showed that in Japan, there is a 97.4% probability that the cost-effectiveness of 12 months of monotherapy is higher than that of 6 months of capecitabine monotherapy.

However, for patients hovering between conventional intravenous chemotherapy and oral capecitabine monotherapy, available studies have confirmed the superiority of adjuvant chemotherapy with intravenous involvement over 6 months of oral monotherapy [5,20]. Despite this, there is no study at this time that confirms the noninferiority of 12 months of oral monotherapy compared to intravenous chemotherapy. Indeed, because of ethical issues, it may not be possible to conduct corresponding randomized controlled trials in the future, but it is undeniable that, based on the results of this study and all other studies, oral capecitabine for 12 months may achieve noninferiority in comparison with intravenous chemotherapy.

## 5. Conclusions

Overall, this study illustrated that for patients with stage III CRC who take capecitabine monotherapy, in the absence of toxicities, extending the oral duration from the basal 6 months to 12 months results in increasing DFS and OS. On the contrary, for patients with high-risk stage II CRC, extending the duration of oral monotherapy does not improve DFS and OS, which means for those who cannot or object to receiving combined chemotherapy, prolonging the monotherapy can be an alternative therapy. However, we are unsure how much survival benefit they may receive from prolonging monotherapy. Therefore, subsequent research will determine how great a survival benefit patients can receive from prolonging monotherapy, and comparing 12 months of monotherapy with combined chemotherapy to gauge whether or not it is non-inferior to 6 months of combined chemotherapy.

## Figures and Tables

**Table 1:** Characteristics of study by duration of adjuvant chemotherapy.

Variables	Total	6 M	12 M	*p*
	(n = 350)	(n = 95)	(n = 255)	
sex, n (%)				0.922
female	147 (42)	39 (41)	108 (42)	
male	203 (58)	56 (59)	147 (58)	
age, n (%)				0.525
≤60	170 (49)	43 (45)	127 (50)	
>60	180 (51)	52 (55)	128 (50)	
location, n (%)				0.569
rectum	260 (74)	68 (72)	192 (75)	
colon	90 (26)	27 (28)	63 (25)	
differentiation, n (%)				0.722
low	99 (28)	26 (27)	73 (29)	
medium	243 (69)	68 (72)	175 (69)	
high	8 (2)	1 (1)	7 (3)	
T (AJCC 8th), n (%)				0.519
T0-3	125 (36)	37 (39)	88 (35)	
T4	225 (64)	58 (61)	167 (65)	
N (AJCC 8th), n (%)				0.378
N0	215 (61)	64 (67)	151 (59)	
N1	104 (30)	24 (25)	80 (31)	
N2	31 (9)	7 (7)	24 (9)	
TNM (AJCC 8th), n (%)				0.204
II (high-risk) ^1^	215 (61)	64 (67)	151 (59)	
III	135 (39)	31 (33)	104 (41)	

^1^ High-risk factors for recurrence (exclusive of those cancers that are MSI-H): poorly differentiated/undifferentiated histology, lymphatic/vascular invasion, bowel obstruction, <12 lymph nodes examined, perineural invasion, localized perforation, or close, indeterminate, positive margins, or tumor budding [4].

## Data Availability

Data are available on request because of privacy restrictions. The data presented in this study are available on request from the corresponding author.
